# Opomorphia

**Published:** 1870-12

**Authors:** 


Opomorphia.—In a communication to the *British Medical Journal,* Dr. F M. Pierce says of this new medicinal agent, that it is obtained by subjecting morphia to the action of pure hydro-chloric acid, at a high temperature, for several hours. In composition opomorphia is morphia, *minus* one atom of water. In its clinical reaction, it is very different from morphia. It is, in some degree, soluble in cold, and much more in warm water. In powder, it is of a snow-white color. Its watery solution is at first colorless, but it soon changes to a dark green, and, in a few weeks, to an almost black color. The nature of this decomposition is not certainly known; morphia, however, is not reproduced. The effect of the colored solution differs from the fresh preparation only in being a little weaker, and slightly more irritating to the skin.

The chief physiological effect of this drug is that of an emetic. So powerful is it in this direction that persons engaged in its manufacture, with all possible precautions against it, absorb sufficient through the skin and lungs to keep them nauseated nearly all of the time they are at work.

One-fifteenth of a grain is a sufficient dose for an adult. He has frequently given one-fortieth of a grain as an emetic to children; he administers it usually subcutaneously, inducing emetics in this way as promptly as when taken by the mouth.

As an emetic, he has used it in pneumonia; to relieve the throat of enudation in diptheria; in scarlatina, in poisoning, and in drunkenness.

As an emetic, it is a depressent, but not to a dangerous extent; sometimes, after emesis, a little drowsiness is observed, but, with sufficiently small doses, this is never a formidable effect. The pulse, respiration, and temperature are not peculiarly effected; neither are the functions of the kidneys or bowels.

Opomorphia is peculiar in the suddenness of its vomiting, the usual premonitory phenomena being almost entirely wanting; generally, there is only one short vomit following a dose, although others may follow.

Dr. Pierce has used it in some cases of cholera, one of which he reports; he gives a dose sufficient for its emetic effect once in two or three days, and has had the pleasure of seeing several of his patients rapidly recover under its use.

But as an emetic, Opomorphia is pre-eminent in the smallness of dose required; the certainty and rapidity of its action; the unimportance of any baneful effect; and its non-irritating character.

It is at present very expensive, but in a short time its more extensive manufacture will doubtless reduce its price so that it may come into more general use.—*Braithwaite.*

